# Epigenetic Reprogramming and Zygotic Genome Activation in Human Preimplantation Development: Mechanisms, Models, and Translational Prospects

**DOI:** 10.1002/rmb2.70095

**Published:** 2026-09-04

**Authors:** Atsushi Fukuda

**Affiliations:** ^1^ Department of Molecular Life Sciences, Division of Basic Medical Science and Molecular Medicine Tokai University School of Medicine Isehara Kanagawa Japan; ^2^ Center for Regenerative Medicine National Center for Child Health and Development Tokyo Japan; ^3^ The Institute of Medical Sciences Tokai University Isehara Kanagawa Japan; ^4^ Micro/Nano Technology Center Tokai University Hiratsuka Kanagawa Japan

## Abstract

**Purpose:**

Early human embryogenesis unfolds through a tightly coupled sequence of events—clearance of maternal transcripts, remodeling of parental chromatin, zygotic genome activation (ZGA), lineage segregation, implantation, and post‐implantation patterning—accompanied by epigenetic reprogramming, including X‐chromosome dosage compensation around the time of implantation. This review aims to synthesize recent advances in understanding this developmental program and to consider their implications for reproductive medicine.

**Methods:**

I review recent literature on human early embryogenesis, with particular emphasis on findings enabled by single‐cell genomics and stem‐cell‐based embryo modeling, and integrate these insights to identify human‐specific features of early development.

**Results:**

These approaches have made previously inaccessible aspects of human early embryogenesis experimentally tractable, revealing molecular and epigenetic features that distinguish human development from that of model organisms, including species‐specific dynamics of ZGA, maternal transcript clearance, chromatin reprogramming, and X‐chromosome dosage compensation.

**Conclusions:**

Advances in single‐cell genomics and embryo modeling are transforming our understanding of human early embryogenesis. Building on these insights, while recognizing their current limitations, I propose a vision for improving reproductive medicine, including the potential for next‐generation embryo selection strategies.

## 1 Introduction

Human embryogenesis during the first two post‐fertilization weeks is a developmental interval of extraordinary compression. Within a few days, a transcriptionally quiescent zygote must transform into a multicellular conceptus with distinct embryonic and extraembryonic lineages, a remodeled epigenome, emergent morphogenetic asymmetries, and the capacity to implant. Although the broad histological sequence has been known for decades, the molecular logic of these events has only recently become accessible through single‐cell transcriptomics, methylomics, chromatin accessibility assays, low‐input histone profiling, and increasingly sophisticated in vitro embryo and stem‐cell‐based models [1, 2, 3, 4, 5, 6, 7, 8, 9, 10, 11, 12, 13, 14].

Among the processes that most strongly organize early human development, zygotic genome activation (ZGA) and X‐chromosome dosage compensation (X‐chromosome inactivation: XCI) occupy a central place because they sit at the intersection of transcriptional control, genome architecture, repeat regulation, and lineage competence. ZGA is not simply the onset of transcription; it is a systems‐level transition that redistributes regulatory authority from gamete‐derived stores to the embryo's own genome. XCI, in turn, is not merely a dosage‐correction mechanism; in humans, it reveals fundamental species differences in how pluripotent and extraembryonic lineages interpret *XIST*, chromatin modifiers, and parent‐of‐origin information [1, 2, 3, 4, 5, 6, 7, 8, 15, 16, 17, 18, 19, 20, 21, 22, 23, 24, 25, 26, 27, 28, 29, 30, 31, 32, 33, 34].

The modern field is also being reshaped by embryo modeling. Human blastoids, 8‐cell‐like cell systems, post‐implantation stem‐cell embryo models, gastruloids, and endometrial coculture platforms now permit mechanistic experiments that were previously impossible in authentic embryos. These systems are already useful for asking which developmental modules can self‐organize, which require maternal tissues, and which remain irreducibly dependent on authentic gametogenesis and fertilization. For reproductive medicine, that distinction is not academic. It determines whether embryo models can become credible platforms for implantation biology, embryo arrest, toxicology, and disease modeling, or whether they remain primarily descriptive analogues [6, 7, 8, 33, 35, 36, 37, 38, 39, 40, 41, 42, 43, 44, 45, 46, 47, 48, 49, 50, 51, 52, 53, 54, 55].

Embryo models are powerful precisely because they make events such as ZGA and lineage specification experimentally tractable. Yet they necessarily begin downstream of the oocyte, whereas in vivo the competence of an embryo is already constrained by the maternal stores it inherits; oocyte quality is therefore as much a determinant of successful preimplantation development as any post‐fertilization event. Seen in this light, ZGA is not an isolated milestone but one step in a coordinated program that begins in the growing oocyte and extends through maternal transcript clearance, epigenetic reprogramming, dosage compensation, and lineage specification. In this review, I follow that program across fertilization—from the mechanisms that allow the oocyte to remain competent for decades, through maternal RNA clearance and ZGA, to epigenetic reprogramming and XCI—and consider how stem‐cell‐based embryo models are clarifying which of its features are human‐specific, and what they may ultimately offer to reproductive medicine.

### 1.1 Unique Features of the Oocyte That Enable Its Long‐Lived State

**FIGURE 1:**
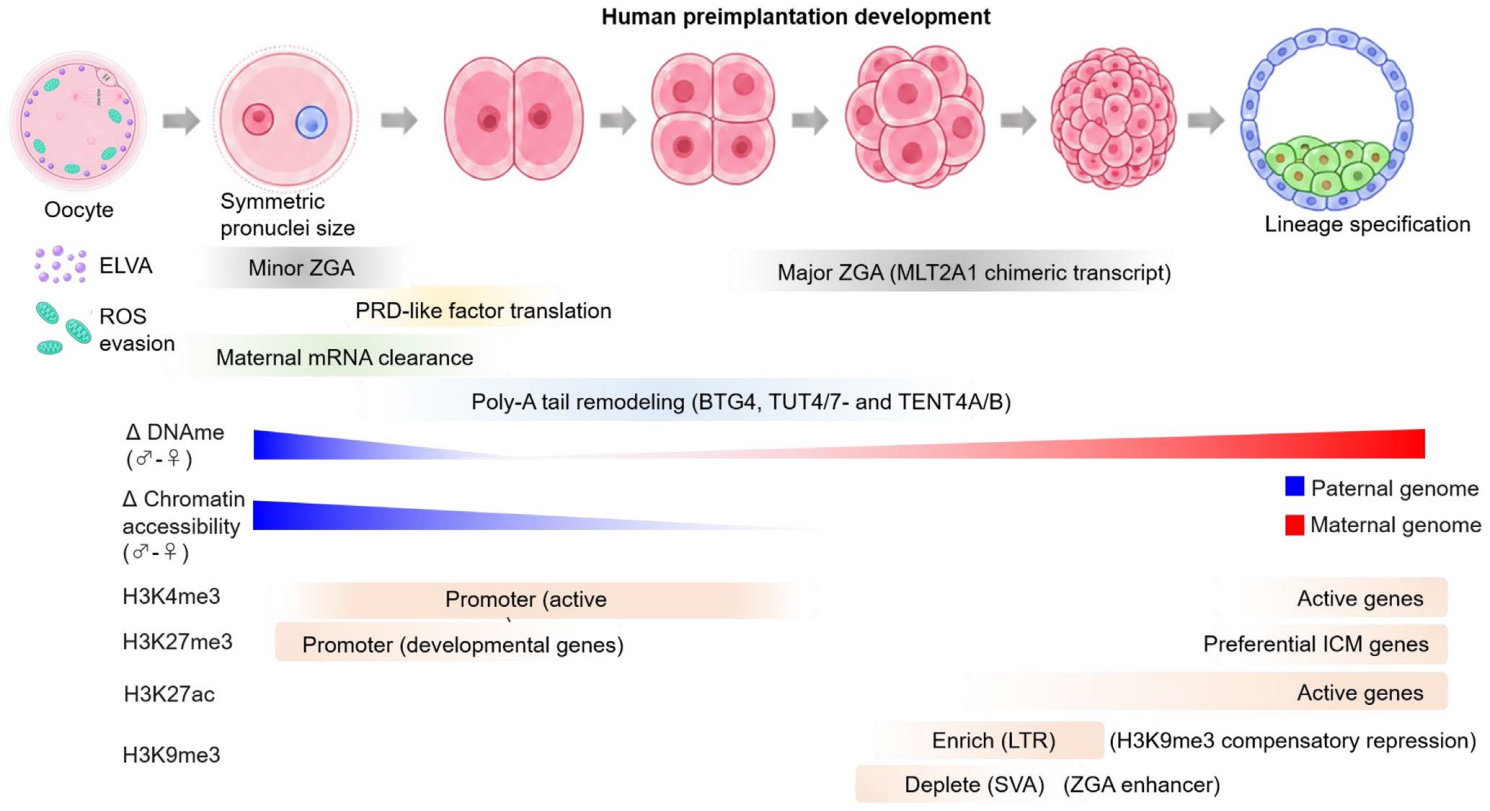
Coordinated program of human preimplantation development: From oocyte competence to lineage specification. Human preimplantation development is depicted from the oocyte and zygote through the two‐cell, four‐cell, and eight‐cell stages to the morula and blastocyst, where initial lineage specification produces the inner cell mass (ICM) and trophectoderm (TE). Developmental competence is established before fertilization: The oocyte must remain viable for decades, and two adaptations that support this long‐lived state are indicated. Endolysosomal vesicular assemblies (ELVAs, purple) sequester damaged and aggregated proteins away from the cytoplasm and are mobilized for degradation shortly before fertilization, preserving proteostasis without constitutive proteolysis. Mitochondria (green) eliminate complex I of the electron transport chain, sustaining metabolic activity while minimizing reactive oxygen species production (ROS evasion). After fertilization, the zygote is characterized by pronuclei of comparable size, in contrast to the pronounced paternal/maternal size asymmetry of the mouse. Two successive phases of zygotic genome activation (ZGA) are indicated: Minor ZGA, which initiates as early as the one‐cell stage; and major ZGA, which peaks at the four‐ to eight‐cell transition, includes expression of MLT2A1‐derived chimeric transcripts originating from endogenous retroelements, and represents the dominant burst of embryonic transcription. Maternal mRNA clearance begins in the zygote and continues through the two‐cell stage, comprising a maternally driven first phase (M‐decay) followed by an embryonically driven second phase (Z‐decay). Concurrent poly(A)‐tail remodeling, mediated by BTG4, TUT4/7, and TENT4A/B, adjusts the translational competence of residual maternal transcripts. During the minor ZGA window, selective translation of PRD‐like homeodomain factors, including TPRXL, TPRX1, and TPRX2, primes the embryo for major ZGA. The parental genomes are asymmetrically reprogrammed during this period. The paternal genome undergoes more extensive and more rapid loss of DNA methylation (ΔDNAme) and chromatin accessibility (ΔChromatin accessibility) relative to the maternal genome, and this parental asymmetry narrows as development proceeds toward the blastocyst stage. Histone modifications follow distinct, stage‐specific trajectories. H3K4me3 and H3K27me3 are both initially promoter‐restricted, but they mark different gene classes and diverge thereafter: H3K4me3 occupies promoters of actively transcribed genes and remains associated with active genes, whereas H3K27me3 occupies promoters of developmental genes, is rapidly lost before major ZGA, and is subsequently re‐enriched at genes associated with ICM identity. H3K27ac, initially broadly distributed, becomes progressively restricted to active regulatory elements as development proceeds. H3K9me3 shows locus‐specific dynamics at retrotransposons: As global DNA methylation declines and can no longer reliably silence repetitive elements, H3K9me3 becomes enriched at long terminal repeat (LTR) elements, acting as a compensatory silencing mechanism that substitutes for the loss of DNA methylation–based repression, whereas H3K9me3 becomes depleted at SINE‐VNTR‐Alu (SVA) elements, where its loss is associated with ZGA‐enhancer activity. By the blastocyst stage, lineage specification is established.

The developmental competence that the embryo inherits is established long before fertilization. Human oocytes are among the longest‐lived cells in the body, formed before birth and remaining viable for several decades, and they must preserve maternal transcripts, proteins, and organelles across that interval. Recent work has revealed unusual homeostatic strategies that make this possible. Dormant oocytes remodel the mitochondrial electron transport chain by eliminating complex I, thereby sustaining metabolic activity while minimizing reactive oxygen species production [56] (Figure 1).

In parallel, oocytes exhibit remarkably low constitutive proteasomal and lysosomal activity and instead sequester damaged or aggregated proteins within endolysosomal vesicular assemblies (ELVAs), which are activated to degrade their cargo shortly before fertilization; failure of this process leads to inheritance of protein aggregates by the embryo and impaired development [57] (Figure 1). These metabolic and proteostatic adaptations underlie oocyte quality and its decline with age, and they define the maternal starting material from which maternal RNA clearance, ZGA, and epigenetic reprogramming subsequently proceed.

### 1.2 Maternal RNA Decay and Minor Zygotic Gene Activation in Human

Human preimplantation development begins with fertilization and pronuclear formation, followed by cleavage divisions, compaction, morula formation, blastulation, and implantation competence. The classic observation that widespread embryonic transcription first becomes obvious between the 4‐cell and 8‐cell stages remains valid at the level of bulk developmental transition, but newer work indicates that the pre‐major‐ZGA interval is more active than previously appreciated. Sensitive transcriptomic analyses have detected minor or preparatory transcription as early as the one‐cell stage (called minor ZGA), implying that the embryo may begin building its own regulatory state before the canonical 8‐cell wave [1, 15, 33, 34].

In mouse zygotes, allele‐aware nascent‐transcription studies and classic nuclear‐transfer/immunofluorescence work indicate that the paternal genome becomes transcriptionally and structurally permissive earlier than the maternal genome. The paternal pronucleus is typically larger, has a more open chromatin configuration, and shows stronger early acetylation/transcriptional activity than the maternal pronucleus. Maternal depletion of Ezh2, a PRC2 catalytic component required for H3K27 methylation, enlarges the maternal pronucleus and disrupts normal preimplantation development, supporting the idea that Polycomb‐dependent maternal chromatin compaction contributes to this parental asymmetry [9, 10, 58, 59]. Whether the same parental bias exists in human zygotes remains unresolved, because allele‐specific nascent‐transcription measurements at the pronuclear stage are still technically limited.

Human zygotes should therefore be discussed cautiously. In conventional clinical embryology, normally fertilized human zygotes are identified by two pronuclei of comparable appearance rather than by a stereotyped large paternal/small maternal asymmetry. Human oocytes also express EZH2 and other chromatin regulators, but expression alone does not demonstrate mouse‐like parental asymmetry [1]. A reasonable working hypothesis is that the more similar pronuclear morphology in human zygotes may give transcriptional regulators broadly comparable access to the maternal and paternal genomes, making early one‐cell transcripts potentially biallelic. This remains a hypothesis, not an established conclusion, and should be tested by allele‐resolved nascent RNA profiling in normally fertilized human zygotes [1, 58, 59, 60].

Single‐cell transcriptomic analyses by Hernandez Mora et al. showed that oocyte‐inherited transcripts are gradually cleared from the human embryo and that failed clearance is associated with developmental arrest [17]. Several conserved maternal‐to‐zygotic transition factors are also expressed during the human oocyte‐to‐cleavage interval, including BTG4 and CNOT6L, which promote deadenylation‐dependent maternal mRNA clearance, and YTHDF2, an m6A reader implicated in maternal transcript decay in mammalian oocytes [61, 62, 63, 64, 65]. These observations support the existence of an active RNA‐clearance program in human embryos, while also emphasizing that direct perturbation evidence in human embryos is the strongest for BTG4.

Maternal RNA decay is usually divided into two mechanistic phases. Maternal decay is driven by factors already stored in the oocyte and can begin before major ZGA, whereas zygotic decay depends on new embryonic transcription after fertilization. In human embryos, these phases are best viewed as overlapping programs rather than sharply separated switches [61, 62, 63, 64, 65].

Sha et al. showed that human maternal transcripts are removed sequentially by M‐decay before major ZGA and Z‐decay after it, and that defects in these processes are common in embryos arrested at the zygote or 8‐cell stages [61]. Just as importantly, their analysis linked failed maternal transcript clearance to clinical embryo arrest, making RNA turnover a mechanistic part of developmental competence rather than a passive cleanup step.

Subsequent human poly(A)‐tail profiling deepened that picture. PAIso‐seq and PAIso‐seq2 analyses showed that during the human oocyte‐to‐embryo transition, maternal transcripts undergo global deadenylation followed by re‐polyadenylation, accompanied by extensive non‐A residue remodeling [62]. In that framework, BTG4 emerges as the best‐supported human maternal “licensing” factor for RNA clearance: BTG4 is highly expressed in human oocytes and early embryos, BTG4 knockdown in human one‐cell embryos shifts poly(A)‐tail distributions toward longer tails, dysregulates hundreds of transcripts, reduces degradation intermediates, and compromises the first cleavage. TUT4/7‐ and TENT4A/B‐dependent remodeling adds another layer, implying that the embryo enters major ZGA with a poly(A)‐coded translational landscape rather than a neutral maternal carryover [62]. This is where the BTG/TTP‐family issue should be handled carefully. In humans, BTG4 has direct perturbation evidence [62]. By contrast, TTP‐family participation—such as ZFP36L2‐like AU‐rich decay logic—is still mostly inferred from pathway conservation, expression, and mammalian precedent rather than from definitive cleavage‐stage human embryo perturbations. Precise timing of maternal mRNA removal therefore appears to be a prerequisite for successful early embryonic development, even where the specific decay factors involved remain incompletely defined.

### 1.3 Molecular Mechanisms for Inducing Major ZGA in Human

One major insight from recent work is that maternal translation is not just supportive housekeeping but a decisive upstream regulator of ZGA. Co‐profiling of translatome and transcriptome across the human oocyte‐to‐embryo transition identified PRD‐like homeobox factors, especially TPRXL, TPRX1, and TPRX2, as highly translated around ZGA [15]. Their depletion impaired ZGA and preimplantation development, while ectopic expression in hESCs activated key ZGA genes [15]. Equally important, this study linked species‐specific translational control to differences in 3′ UTR architecture. In human oocytes, PAS‐proximal cytoplasmic polyadenylation elements in transcripts such as OTX2 and TPRXL are associated with CPEB1‐dependent translational activation during maturation, increasing the abundance of proteins that help initiate major ZGA [15].

These findings complicate the longstanding emphasis on DUX4 as the dominant upstream human ZGA regulator [66, 67]. Mouse Dux and human DUX4 can activate cleavage‐stage genes and ERVL/HERVL retrotransposons in cultured cells, but mouse knockout studies showed that loss of Dux causes weaker or more context‐dependent developmental effects than would be expected for a single master regulator. In human embryos, DUX4 repression also produces limited global transcriptional change, although DUX4 can prime subsets of ZGA and repeat‐associated genes. A balanced model is therefore that DUX4 contributes to a broader ZGA network rather than acting as the sole upstream trigger [11, 66, 67, 68, 69, 70].

A second major insight is that human ZGA exploits primate‐specific retroelement‐derived mechanisms [71, 72, 73]. Recent study showed that MLT2A1, an ERVL‐derived LTR family strongly induced during ZGA, is selectively reduced in 8‐cell‐arrested in vitro fertilization (IVF) embryos and functionally required for normal development [16]. Mechanistically, MLT2A1 copies generate heterologous chimeric RNAs with diverse downstream sequences. The variable 3′ fused portions appear to broaden genomic targeting, while the conserved 5′ MLT2A1 component binds HNRNPU and promotes RNA polymerase II recruitment. This is more than a correlative retroelement signal: it is a mechanistic example of transposable‐element exaptation into a distributed transcriptional activation network [7, 16, 73].

These recent studies indicate that coordinated transcription‐factor activity and regulated repeat expression are essential for normal preimplantation development. Yet successful ZGA does not guarantee implantation. Even after transfer of embryos selected by morphology and, in some settings, preimplantation genetic test for aneuploidy (PGT‐A), a substantial fraction of euploid blastocysts fail to implant or do not progress to live birth [74]. This clinical gap indicates that chromosome copy number is necessary but not sufficient as a predictor of developmental competence; molecular readouts of ZGA quality, epigenetic resetting, lineage allocation, and implantation readiness will be needed to improve embryo selection [17, 33, 53, 74, 75].

### 1.4 Epigenetic Dynamics During ZGA and Early Preimplantation Embryos

Human preimplantation development is not an epigenetic “blank‐slate” interval, but a tightly ordered reset in which DNA methylation, chromatin accessibility, and histone H3 modifications are erased, preserved, or rebuilt with strong stage specificity. In the human embryo, the major logic of this reset is to make ZGA possible without losing imprinting, genome stability, or the capacity to segregate lineages a few days later; in practice, this means widespread DNA demethylation, highly selective retention of some methylated loci, and rapid remodeling of H3K4me3, H3K27me3, and H3K9me3 around promoters, enhancers, and repeats [33, 76, 77, 78, 79, 80]. The sections below examine these DNA methylation, chromatin‐accessibility, and histone H3 reprogramming events in turn (Figure 1).

### 1.5 DNA Methylation Dynamics and Function

DNA methylation in mammals is chiefly 5‐methylcytosine at CpG dinucleotides [81]. In biochemical terms, DNMT3A and DNMT3B are the principal de novo methyltransferases, DNMT3L is a catalytically inactive cofactor that strengthens establishment in the germline, and DNMT1, together with UHRF1, maintains methylation across DNA replication [33, 34]. Removal can occur passively, when maintenance fails, or actively through oxidation of 5mC by TET enzymes, notably TET3 in the oocyte‐to‐zygote interval in mouse and plausibly in human as well [12, 33, 82].

Human preimplantation embryos then undergo global but selective DNA methylation erasure. Whole‐genome and single‐cell bisulfite‐based studies showed a major demethylation wave within roughly the first 12 h after fertilization, affecting especially intergenic and enhancer‐rich regions and many gene‐body regions, followed by additional loss from late zygote to 2‐cell and again from 8‐cell to morula/blastocyst stages [76, 77, 78, 79]. Importantly, this is not indiscriminate erasure. Some imprinting control regions and subsets of transposons resist complete demethylation, and those protected loci are biologically consequential because they preserve parent‐of‐origin information and suppress potentially harmful repetitive elements. Multi‐omics approaches at single cell resolution using human preimplantation embryos reveal that paternal DNA methylation occurs earlier, prior to the 2‐cell stage, than that of the maternal genome, whereas the maternal genome becomes extensively demethylated from around the 4‐cell stage onward [4].

The mechanism of this demethylation in human embryos remains less settled than in mouse. Mouse work established a prominent role for maternal TET3 in active paternal‐genome demethylation immediately after fertilization, but in human embryos the balance between active oxidation and passive replication‐coupled dilution is still debated [12, 82]. Human zygote/cleavage‐stage immunostaining for 5mC and 5hmC supports oxidative turnover, and ultrasensitive translatome profiling showed that TET3 is among maternal transcripts translationally activated during the human oocyte‐to‐embryo transition [15], making an active component plausible. At the same time, the overall methylome trajectories are equally compatible with incompletely maintained methylation across rapid cleavage divisions [4, 12, 15, 33].

A second key point is that DNA methylation is not merely erased; there is also transient de novo methylation. Single‐cell BS‐seq studies found that from 4‐cell to 8‐cell, newly methylated regions are enriched at SINEs, LINEs, and LTRs, after which much of this methylation is lost again by morula/blastocyst stages [4, 33]. This transient behavior is analytically important because it argues against the simplistic view that low methylation alone defines the preimplantation state. Instead, the embryo appears to use DNA methylation dynamically as a temporary constraint on genome instability while major ZGA permits highly unusual bursts of transcription, including regulated retroelement expression.

The importance of proper methylome reprogramming is underscored by developmental failure states. Using scM&T‐seq, which combines single‐cell RNA‐seq with PBAT‐based scBS‐seq from the same cell, Hernández Mora et al. [17] showed that developmentally arrested cleavage‐stage embryos display defective EGA together with abnormal DNA methylation dynamics. In those arrested embryos, methylation remained inappropriately high and was associated with perturbed expression of maintenance machinery including DNMT1 and UHRF1 [17]. That study does not prove a single causal methylation lesion, but it strongly supports the broader conclusion that methylome clearance is not a passive backdrop; it is part of the competence program that allows human embryos to survive the maternal‐to‐zygotic transition.

### 1.6 Histone H3 Remodeling and ZGA Competence

The histone H3 landscape is even more stage‐specific than the methylome, and it is here that several major human‐versus‐mouse differences become especially important. Mechanistically, the most informative H3 marks in human preimplantation embryos are H3K4me3, H3K27me3, and H3K9me3, with H3K27ac providing an important complementary readout of active regulatory DNA [33, 83].

Recent advances such as ultra‐low‐input CUT&RUN and single‐cell multiomic profiling have enabled high‐resolution mapping of these transitions in human embryos, revealing a regulatory architecture distinct from that of other mammals [80]. In human oocytes, the permissive histone mark H3K4me3 is predominantly arranged in canonical, promoter‐restricted peaks, contrasting sharply with the broad non‐canonical H3K4me3 domains observed in mouse oocytes [80]. This indicates that human oocytes do not rely on widespread H3K4me3 to maintain transcriptional competence. Instead, the major chromatin remodeling event occurs after fertilization, during the minor ZGA phase. At this stage, human embryos acquire a globally permissive chromatin state, marked by widespread H3K4me3 deposition across CpG‐rich promoters and regulatory elements. This expansion of H3K4me3 effectively primes the embryonic genome for subsequent activation, establishing a chromatin environment conducive to large‐scale transcriptional initiation.

Concurrently, the repressive mark H3K27me3 undergoes rapid and global depletion immediately after fertilization [80]. This depletion removes parental epigenetic constraints and facilitates the transition to a permissive chromatin state. The loss of H3K27me3 is particularly notable because it occurs genome‐wide, including at developmental regulators that will later require precise repression. As embryos progress toward major ZGA, CpG‐rich regulatory regions diverge into active and repressed states. Active regions retain H3K4me3 and gain additional activating marks, whereas repressed regions gradually reacquire H3K27me3, restoring developmental control. This post‐ZGA restoration of H3K27me3 is essential for establishing lineage‐specific transcriptional programs and restricting inappropriate gene activation [80].

Integration of chromatin and transcriptome maps further reveals that early lineage segregation in human embryos is accompanied by asymmetric H3K27me3 patterning, particularly between the inner cell mass (ICM) and trophectoderm (TE). ICM‐specific genes exhibit distinct H3K27me3 dynamics that help establish pluripotent identity, whereas TE‐specific genes do not show comparable asymmetry [80]. These findings highlight a unique mode of epigenetic regulation in which H3K27me3 asymmetry contributes to early lineage decisions.

Collectively, these observations define a priming phase that bridges parental and zygotic epigenomes in humans. This phase is characterized by post‐fertilization H3K4me3 expansion, global H3K27me3 depletion, and subsequent lineage‐specific restoration of repressive marks. Together, these processes establish a permissive yet tightly regulated chromatin landscape that enables human embryos to transition from parental control to autonomous developmental programming.

H3K27ac, a hallmark of active promoters and enhancers, undergoes extensive and stage‐specific remodeling during human preimplantation development [80, 83]. In human oocytes, H3K27ac levels are generally low, reflecting minimal enhancer activity and transcriptional quiescence. After fertilization, early cleavage‐stage embryos (2‐cell and 4‐cell) exhibit broad H3K27ac domains distributed genome‐wide, a pattern conserved across mammals and thought to support a permissive chromatin environment before minor ZGA. These broad acetylation domains frequently overlap with broad H3K4me3 regions, suggesting coordinated priming of regulatory elements prior to transcriptional onset.

At the 8‐cell stage, coinciding with major ZGA in humans, broad H3K27ac domains transition into sharp, typical peaks at promoters and distal enhancers. This shift marks the emergence of stage‐specific enhancer activity and correlates with activation of embryonic transcriptional programs. P300/CBP acetyltransferases play essential roles in establishing these H3K27ac peaks, and their inhibition disrupts enhancer function and gene expression [80]. After ZGA, H3K27ac becomes increasingly lineage‐specific: inner cell mass (ICM) and trophectoderm (TE) acquire distinct enhancer landscapes, with TE showing particularly strong conservation of H3K27ac‐marked distal enhancers across species. These enhancers participate in long‐range chromatin interactions and help drive early lineage segregation.

Together, these findings indicate that human embryos undergo a unique sequence of chromatin remodeling in which broad H3K27ac domains transition into precise enhancer‐linked acetylation, enabling coordinated activation of developmental gene networks.

The dynamics of H3K9me3 in human preimplantation embryos have long remained elusive due to technical and ethical constraints on embryo research. Two recent studies, Xu et al. [84] and Yu et al. [85], addressed this gap using low‐input epigenomic profiling techniques—Cut&Run and ultra‐low‐input native ChIP‐seq, respectively—applied directly to human embryos. Both groups revealed that H3K9me3 behaves strikingly differently from other histone marks during this period. While H3K27me3 and H3K4me3 undergo dramatic resetting to their lowest levels at the 8‐cell (8C) stage, H3K9me3 instead increases continuously starting after fertilization and continues rising past the 8C stage. Both teams found that H3K9me3 is deposited on retrotransposons and regulates their expression, with some elements repressed throughout development and others regulated in a stage‐specific manner.

Xu et al. [84] demonstrated stage‐specific H3K9me3 deposition on distinct classes of LTR retrotransposons, often substituting for the rapidly resetting DNA methylation, and showed—using a Dux knockout mouse model—that Dux is required for proper establishment of H3K9me3 domains. Yu et al. [85], meanwhile, screening for putative 8C‐stage enhancers, identified a set of hominoid‐specific SINE‐VNTR‐Alu (SVA) elements on which H3K9me3 deposition decreases from the 4‐cell to 8C stage. This reduction facilitated SVA interaction with ZGA genes, and H3K9me3 levels correlated negatively with ZGA gene expression. Because these SVA elements harbor binding motifs for Dux, a known ZGA inducer, but are suggested to be a non‐essential factor for early development [11], the authors proposed that SVAs act as mediators of ZGA. CRISPR‐KRAB‐mediated silencing of SVA_D repeats confirmed this, producing defective ZGA and reduced blastocyst formation rates [85].

Beyond ZGA, both studies implicated H3K9me3 in lineage segregation between the inner cell mass and trophectoderm, and in the evolutionary “arms race” between KRAB‐ZNF proteins and retrotransposons. Together, these findings position H3K9me3‐regulated, species‐specific retrotransposons as key contributors to the evolution of human‐specific developmental programs.

### 1.7 X‐Chromosome Inactivation Timing and Mechanisms in Human Embryos

Human X‐chromosome dosage compensation (X‐chromosome inactivation: XCI) begins from a state that differs substantially from mouse. In female human preimplantation embryos, both X chromosomes are generally active, and XIST can be expressed from active X chromosomes rather than marking a fully inactive X chromosome (Xi); male embryos, too, express XIST from their single, active X chromosome during this period [18, 19, 20, 21, 22, 23, 24, 25, 26, 27, 28, 29, 30, 31, 32, 33]. This uncoupling of XIST expression from chromosome‐wide silencing is one of the central species‐specific features of early human development.

The preimplantation literature remains divided between at least two interpretations. One proposes X‐chromosome dampening, meaning that expression from both active X chromosomes is gradually reduced without classic full inactivation [21, 30]. Another proposes progressive XCI affecting subsets of genes or cells by blastocyst stages. The disagreement partly reflects analytical choices, stage sampling, and the difficulty of inferring chromosome‐wide states from sparse single‐cell allelic data [21]. The most defensible synthesis at present is therefore conservative: female human blastocysts exhibit active‐X‐associated XIST expression and partial dosage‐adjustment phenomena, but they do not yet show a completed somatic‐like XCI program [21, 22, 23, 24, 25, 26, 27, 28, 29, 30, 31, 32, 33].

An interesting observation that precedes stable XCI establishment comes from human naive PSC models, which approximate the pre‐X‐inactivation female epiblast state [32]. In these systems, XIST has direct silencing capacity in trans as well as in cis [86]. Whether this trans‐repressive activity also operates in the embryo itself before implantation has not yet been tested directly, so its in vivo functional significance remains unknown.

Returning to the embryos themselves, in vitro cultured human embryos analyzed through implantation‐stage development show increasing allelic skewing of X‐linked expression from around Day 8 to Day 10, consistent with the onset of random XCI, and suggest that inactivation remains incomplete even by Day 12 [18]. Comparative primate data align well with this interpretation and further indicate lineage‐specific kinetics, with trophoblast inactivating faster than epiblast and hypoblast [25]. That lineage asymmetry is likely to become highly relevant for interpreting trophoblast stem cells, blastoids, and implantation models [18, 25, 26, 27, 28, 29, 30, 33, 53].

**FIGURE 2:**
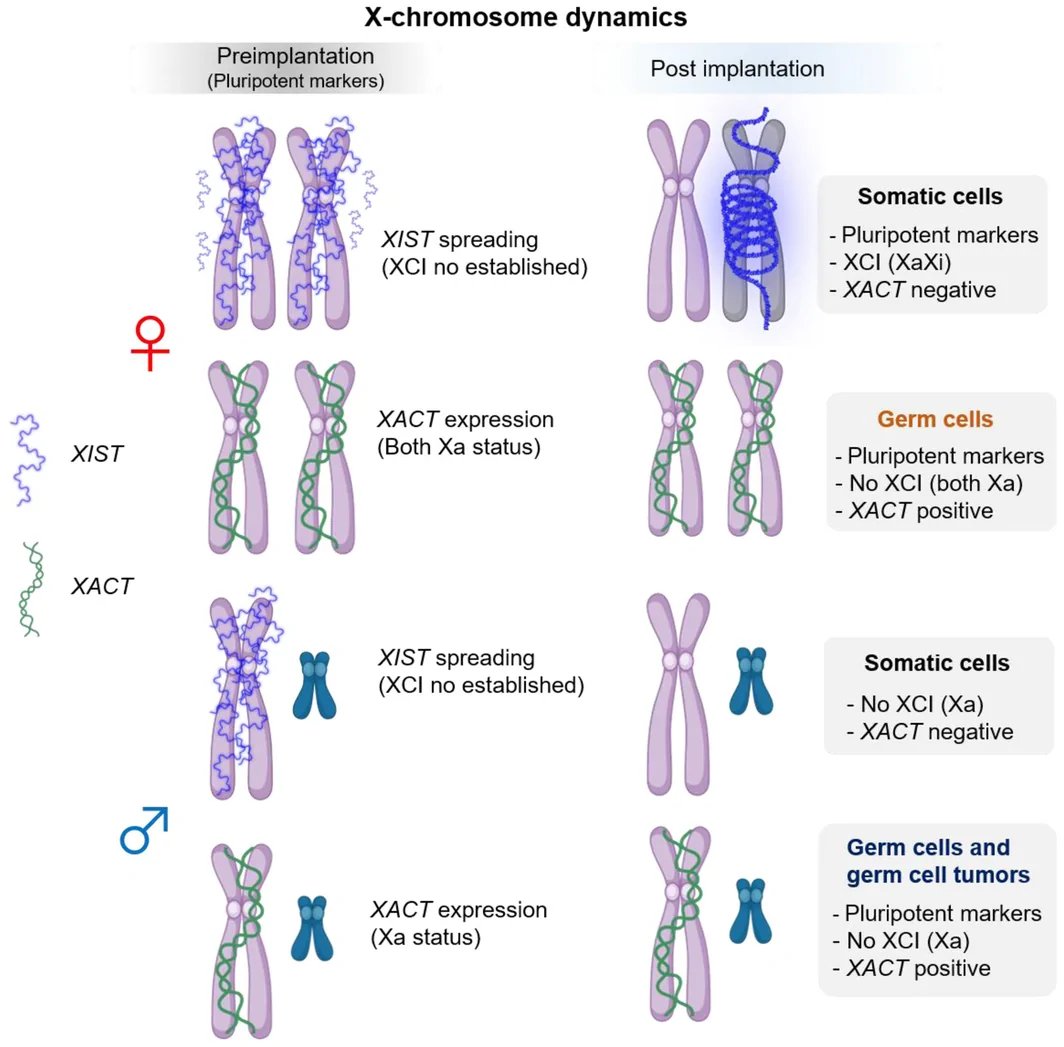
*XIST* and *XACT* dynamics in human embryos, germ cells, and somatic cells across developmental stages. Schematic illustrating X‐chromosome status in female (♀, upper two rows) and male (♂, lower two rows) cells at preimplantation and post‐implantation stages. Purple wavy lines denote *XIST* transcripts; green wavy lines denote *XACT* transcripts. Darker chromosome shading indicates progression toward X‐chromosome inactivation (XCI). In female preimplantation embryos, *XIST* spreads biallelically across both X chromosomes without establishing stable XCI, while *XACT* is co‐expressed and coats the active X chromosome (Xa) in the context of pluripotency‐associated markers. After implantation, female somatic cells exhibit canonical random XCI (one Xa and one inactive X: Xi), with loss of pluripotent markers and *XACT* negativity. In contrast, female germ cells retain both X chromosomes in an active state (Xa/Xa), maintain pluripotent markers, and remain XACT‐positive, consistent with XACT's association with the pluripotent state rather than with XCI per se. In male preimplantation embryos, *XIST* spreads across the single X chromosome without inducing dosage compensation, and *XACT* is expressed. Post‐implantation male somatic cells carry a single Xa and become *XACT*‐negative, whereas male germ cells and germ cell tumors retain pluripotent markers and *XACT* positivity. Together, this diagram highlights that *XACT* expression tracks pluripotency rather than XCI status, and that stable random XCI in females is a post‐implantation event.

Mechanistically, much remains unresolved around XCI establishment. In mice, Xist regulation depends on a multilayered X‐inactivation center that includes Tsix, the antisense lncRNA that represses Xist in cis, and dosage‐sensitive activators such as RNF12/RLIM [26]. RNF12 promotes Xist upregulation in part through degradation of REX1/ZFP42, thereby relieving repression of Xist [26]. Human preimplantation embryos differ substantially from this mouse model: they do not show a simple imprinted XIST pattern, and a canonical TSIX‐equivalent antisense transcript has not been demonstrated. Instead, hPSCs and human preimplantation embryos express XACT, a lncRNA that coats the active X chromosome [24, 31, 87]. XACT can antagonize aspects of XIST biology, but XACT deletion in hPSCs does not produce a simple X‐linked dosage phenotype; reported effects are more closely linked to pluripotency and neural differentiation [88, 89]. Moreover, XACT expression is also retained in early primordial germ cells in humans [90], which also show NANOG expression, consistent with the notion that XACT expression is positively correlated with the pluripotency network (Figure 2).

How XIST is induced in the human embryo remains unknown. Because XIST expression appears around the major ZGA window, can be biallelic in female embryos, and is detectable even from the single X chromosome in some male embryos, its initial activation may reflect chromatin opening and permissive transcription during ZGA rather than the counting‐dependent choice mechanism that later stabilizes random XCI [1, 19, 20, 21, 22, 23, 24, 33].

Another unresolved question is how X‐chromosome counting occurs. In both mouse and human, stable dosage compensation is a post‐implantation process, but the initiating logic differs across species. In humans, the critical counting‐related event may not be the first activation of XIST itself, because early XIST expression is not restricted to one allele. Rather, counting may correspond to the resolution of an initially permissive biallelic XIST state into distinct inactive and active X‐chromosome identities. Under this model, stochastic differences in XIST accumulation between homologous X chromosomes could be amplified through cis‐acting feedback mechanisms. The chromosome exhibiting higher XIST levels would preferentially recruit silencing factors such as SPEN, Polycomb complexes, and associated chromatin modifiers, leading to progressive repression of X‐linked genes and stabilization of the inactive X state [19, 20, 21, 22, 23, 24, 25, 26, 27, 28, 29, 30]; the chromosome with lower XIST expression would correspondingly fail to recruit silencing machinery efficiently and would remain transcriptionally active. On this view, human X‐chromosome counting is best understood as a mechanism that restricts productive, chromosome‐wide silencing to a single X chromosome, rather than one that determines whether *XIST* is activated in the first place.

### 1.8 Human Embryo Models for Post‐Implantation Development

Despite being essential for establishing a viable pregnancy, the biological mechanisms that govern human embryo implantation and placental development remain insufficiently characterized. This gap is particularly consequential because unsuccessful implantation is a leading cause of early pregnancy loss in both natural conception and in vitro fertilization, with fewer than 30% of human embryos achieving successful implantation into the endometrium [91]. This vulnerability is compounded by pregnancy complications such as preeclampsia, a hypertensive disorder affecting 2%–8% of pregnancies worldwide that is thought to arise in part from impaired trophoblast invasion into the maternal decidua during the first trimester [91]. These clinical observations underscore the importance of elucidating the molecular and cellular processes that govern trophoblast lineage specification, tissue invasion, and maternal‐fetal communication.

To help close this knowledge gap, recent efforts have focused on developing 3D in vitro platforms that simulate the human implantation microenvironment. One notable example combines human‐pluripotent‐stem‐cell‐derived blastoids with hormonally primed endometrial assembloids in a 3D in‐chip implantation model; using this system, Li and colleagues identified compounds that markedly improved implantation efficiency in endometrioids derived from patients with recurrent implantation failure [92].

Recent studies indicate that blastoids are powerful tools for implantation biology. Several approaches have been developed to generate human blastocyst‐like structures, most commonly from naive or reset pluripotent stem cells whose transcriptional state resembles early embryonic stages rather than conventional primed epiblast [32, 40, 41]. Among these, 4CL blastoids show blastocyst‐like morphology and transcriptional profiles, and have been reported to preserve important aspects of DNA methylation and imprinting [93]. Extended three‐dimensional culture can support progression toward early post‐implantation or gastrulation‐like states, making blastoids useful for testing how preimplantation lineage allocation is coupled to implantation‐stage morphogenesis. These models nevertheless remain approximations and require benchmarking against authentic embryos, especially for imprinting, X‐chromosome state, trophoblast maturation, and extraembryonic lineage fidelity.

Because direct gene‐functional assays in human embryos are difficult, embryo modeling should be integrated with comprehensive perturbation platforms such as CRISPR inhibition, CRISPR activation, and single‐cell Perturb‐seq [94, 95]. This combination would make it possible to identify genes that control human‐specific ZGA, lineage allocation, trophoblast invasion, and post‐implantation patterning in scalable in vitro systems. The most informative studies will pair perturbation with single‐cell transcriptomics, chromatin accessibility, methylation, and copy‐number inference, because embryo models are heterogeneous and developmental competence is unlikely to be captured by one molecular layer alone.

### 1.9 Applications to Assisted Reproductive Technologies, Translational Gaps, and Future Perspectives

The clearest translational application of early‐human‐embryo biology is deeper interpretation of embryo competence. Current IVF practice still relies heavily on morphology, blastocyst development, and selective use of add‐ons such as PGT‐A or time‐lapse imaging [96]. Yet developmental competence emerges from coordinated execution of ZGA, epigenetic resetting, lineage allocation, repeat control, and implantation signaling. The next generation of embryo assessment should therefore be multiparameter rather than single‐test based, combining morphokinetics, chromosome copy‐number status, sequence‐level variants, noninvasive transcript fragments, methylation or chromatin‐derived markers, and model‐informed perturbational signatures.

PGT‐A illustrates both the power and the limits of current translation. Aneuploidy is unquestionably common in human embryos and biologically important, but the embryo is also frequently mosaic, and biopsy‐based inference from a small trophectoderm sample may not reflect the whole conceptus. Regulatory and professional bodies remain cautious about claims that PGT‐A improves live birth for most fertility patients, while acknowledging that it may reduce miscarriage or shorten time to pregnancy in selected contexts. Developmentally, this is unsurprising: chromosome number matters, but successful implantation also depends on ZGA quality, epigenetic competence, lineage organization, and the ability of mosaic or stressed embryos to compensate [16, 84].

An important and still underappreciated question is what drives chromosomal instability and mosaicism in the first place. Beyond DNA damage itself, recent work indicates that the temporal coordination among DNA replication, DNA repair, and cell‐cycle progression is a major determinant of genome stability in cleavage‐stage embryos. Single‐cell replication‐timing analyses show that the replication program is itself developmentally regulated and that its establishment coincides with a window of heightened fragility. In mouse embryos, a somatic‐like replication‐timing program emerges abruptly at the 2‐to‐4‐cell transition, coincident with strengthening of nuclear compartments but uncoordinated with fork‐speed regulation; the resulting transient replication stress and under‐replication are accompanied by a marked increase in chromosome breaks and segregation errors at the 4‐cell stage [97]. Complementary analyses indicate that late‐replicating regions associate with the nuclear lamina, contain few active or dormant origins, and are preferentially targeted by spontaneous chromosome breaks, including at sites concordant between bovine and human embryos [98]. Although the precise stage at which replication timing is first established differs between these studies, both link the emergence of the replication program to a window of elevated genome instability. Importantly, these segregation errors were rescued by nucleoside supplementation of the culture medium, which accelerated replication fork speed and relieved replication stress in mouse embryos [97]. Because human cleavage‐stage embryos are routinely cultured in vitro, this raises the possibility that comparable modification of culture conditions could reduce replication‐associated aneuploidy and mosaicism in human embryos, although this remains to be tested directly.

Early human embryos also appear repair‐deficient: Cas9‐induced double‐strand breaks frequently generate aneuploidy and large deletions, whereas base editors, which avoid double‐strand breaks, support normal development to the blastocyst stage without chromosomal alterations [99]. Together these findings suggest that mosaicism and segmental aneuploidy arise not from purely stochastic mitotic errors but from structurally predisposed genomic regions encountering a transiently permissive replication‐repair environment.

Time‐lapse imaging provides a parallel lesson. The biological rationale is strong: continuous observation can capture cleavage synchrony, compaction dynamics, and abnormal transitions that snapshot morphology misses. However, current evidence has not established that time‐lapse selection improves live birth for most patients. The implication is not that morphokinetic information lacks biological meaning, but that current translation from developmental signal to clinically validated decision rule remains incomplete. A recent study has extended this rationale by introducing a minimally invasive live‐imaging platform for long‐term, high‐resolution visualization of human blastocysts. Combining optimized mRNA electroporation for nuclear labeling with light‐sheet microscopy, Abdelbaki et al. [100] directly captured chromosome missegregation—including multipolar spindle formation, lagging chromosomes, and mitotic slippage—immediately before implantation, and showed that most lagging chromosomes are passively inherited rather than reincorporated, demonstrating that mosaic aneuploidy can arise de novo during late preimplantation development. Deep‐learning‐based 3D segmentation further showed that most labeled cells remain externally positioned, consistent with a trophectoderm rather than an inner cell mass fate [100]. These findings refine our understanding of human developmental dynamics and highlight an important limitation in interpreting PGT‐A results, because late‐stage mitotic errors may be confined to the placental lineage.

Embryo models may help close this gap by providing testbeds for implantation biology, trophoblast dysfunction, recurrent implantation failure, endocrine defects, developmental toxicology, and embryo‐endometrium communication. The endometrial co‐culture and scaffold systems are especially promising because they permit direct study of embryo‐maternal signaling rather than embryo‐in‐isolation phenotypes. Still, before these models can guide clinical decision‐making, the field will need clearer benchmarks of fidelity, standardized comparators to authentic embryos, and prospective evidence that model‐derived biomarkers improve patient outcomes rather than merely correlate with them [33, 53, 54, 55].

**FIGURE 3:**
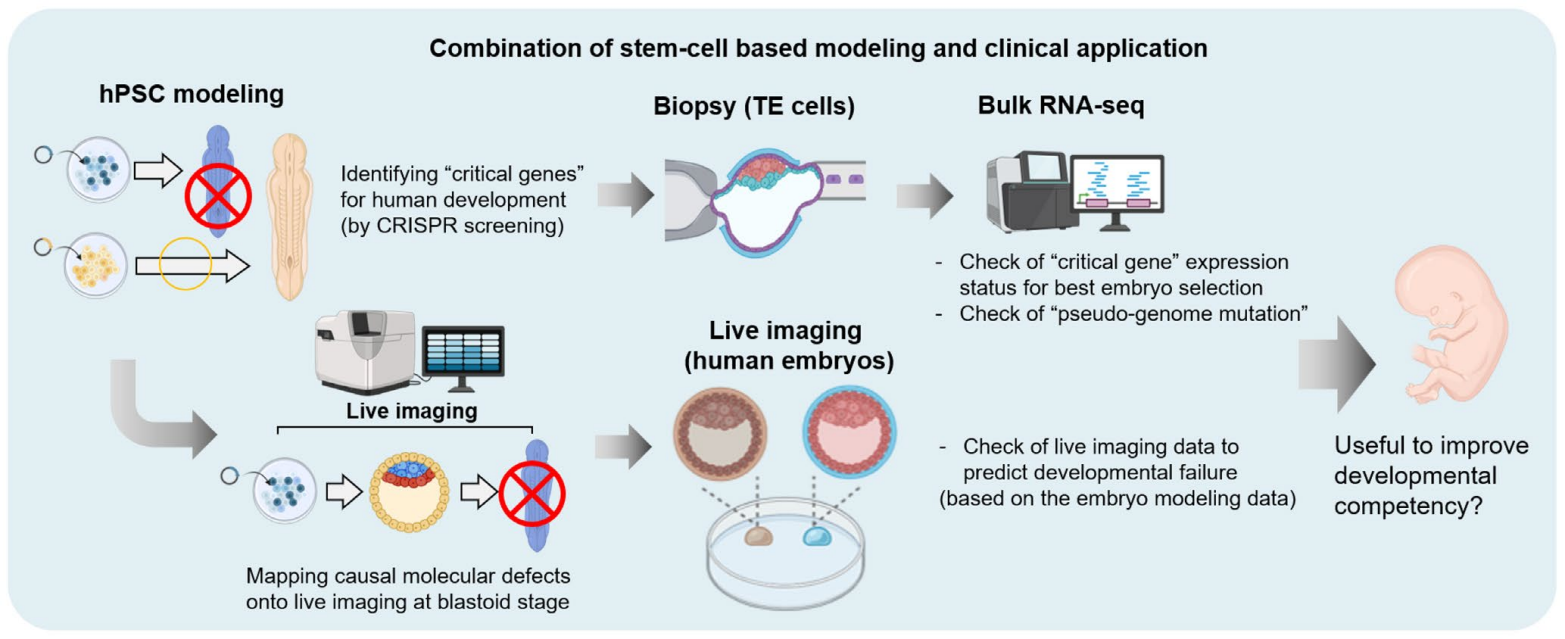
Future perspective: Combining stem‐cell‐based embryo modeling with clinical embryo assessment. A conceptual framework in which mechanistic insight obtained invasively in human pluripotent stem cell (hPSC)–based embryo models is translated into noninvasive or minimally invasive readouts for clinical embryo assessment. Upper path: HPSC‐based models carrying targeted perturbations (CRISPR screening, knockout/knockdown, or overexpression) are used to identify the critical genes and pathways governing human preimplantation development, including ZGA, lineage specification, and epigenetic reprogramming. The resulting gene set is then queried in IVF embryos by bulk RNA sequencing of trophectoderm (TE) cells obtained through the biopsy procedure already used for preimplantation genetic testing, allowing both the expression status of these critical genes and putative pseudo‐genome mutations—sequence variants that may impair developmental gene function without meeting conventional criteria for pathogenicity—to be assessed. Lower path: The same perturbed models are followed by live imaging to the blastoid stage, so that each defined molecular defect can be mapped onto the morphological and morphokinetic signature it produces. This model‐derived catalog of causal defect‐to‐phenotype relationships provides a reference against which live imaging of patient embryos can be interpreted, offering a principled basis for predicting developmental failure from imaging data alone. Right: The two paths provide independent but complementary routes toward improved developmental competence. The upper path acts at the molecular level, distinguishing chromosomal normality from true developmental competence, whereas the lower path acts at the imaging level, allowing developmental failure to be anticipated noninvasively. Either path alone, or the two used together, is proposed as a means of selecting embryos with the highest developmental competence, with the ultimate aim of improving implantation and live‐birth outcomes.

The major gap is translational. A promising new approach uses transcriptome‐based embryo selection to integrate genome variation with transcriptional state and developmental modeling. A practical future workflow could combine trophectoderm copy‐number and sequence information, noninvasive RNA‐derived signatures, and embryo‐model‐based perturbation data to identify euploid embryos whose ZGA, lineage, or implantation programs are at risk. This approach would aim to distinguish chromosomal normality from true developmental competence, but it must be prospectively validated against implantation, miscarriage, placentation, and live‐birth outcomes (Figure 3).

Another direction is to use embryo models to link molecular causation to noninvasive readouts. Because stem‐cell‐based embryo models are amenable to gene editing and other targeted perturbations, the molecular requirements for successful ZGA, lineage specification, and implantation can be tested directly, and the resulting phenotypes characterized in depth by transcriptomic, epigenomic, proteomic, and metabolomic profiling. Live imaging of the same perturbed models can then reveal which morphological and morphokinetic signatures accompany each defined molecular lesion. Mapping causal molecular defects onto imaging phenotypes in this way would provide a principled basis for recognizing impending developmental failure from time‐lapse data alone, allowing mechanistic insight obtained invasively in embryo models to be applied noninvasively to patient embryos. Such model‐anchored integration, rather than any single assay, is likely to represent the most realistic route toward precise and minimally invasive embryo assessment (Figure 3).

Viewed as a whole, embryo competence is best understood as the coordinated execution of several interdependent modules—oocyte quality, maternal RNA clearance, ZGA, epigenetic reprogramming, X‐chromosome dosage compensation, lineage allocation, and implantation—rather than as any single measurable parameter. Progress in human reproductive medicine will therefore likely depend less on refining individual tests than on understanding how these modules are coupled and how their coupling fails.

## Funding

This study was supported by JSPS KAKENHI Grant Number 23H00436 to A.F.

## Conflicts of Interest

The author declares no conflicts of interest.

## Data Availability

Data sharing was not applicable to this article as no datasets were generated or analysed during this study.
